# The therapeutic potential of glucagon-like peptide-1 receptor analogs for neuroinflammation in the setting of asthma

**DOI:** 10.37349/eaa.2025.100967

**Published:** 2024-12-17

**Authors:** Courtney Lehman, Ray Stokes Peebles

**Affiliations:** 1 Division of Allergy, Pulmonary, and Critical Care Medicine, Vanderbilt University Medical Center, Nashville, TN 37232, USA; 2 Department of Pathology, Microbiology, and Immunology, Vanderbilt University School of Medicine, Nashville, TN 37232, USA; 3 United States Department of Veterans Affairs, Nashville, TN 37215, USA

**Keywords:** Glucagon-like peptide-1 receptor, glucagon-like peptide-1 receptor agonist, asthma, neuroinflammation

## Abstract

Glucagon-like peptide-1 (GLP-1) is a hormone that regulates blood glucose levels and is produced by the enteroendocrine glands in the large and small intestines in response to the consumption of foods that contain carbohydrates, fats, and proteins. When GLP-1 is secreted, it acts on the pancreas to increase insulin production and secretion, while decreasing pancreatic glucagon secretion in order to lower serum glucose. However, GLP-1 also regulates metabolism through the gut-brain axis. While GLP-1 is primarily produced in the gut and released into the bloodstream, small quantities of it can also be synthesized in distinct areas of neurons located in the hindbrain. Recent studies have proposed that GLP-1 receptor (GLP-1R) agonists (GLP-1RAs) may protect against neuroinflammatory diseases. GLP-1RAs may also be a therapeutic target for asthma as animal models show that these drugs reduce allergen-induced airway inflammation, as the GLP-1R is expressed on lung epithelial and endothelial cells. There is a notable association between insulin resistance and the onset of asthma, particularly among obese people, with this association suggesting that metabolic dysfunction may play a role in asthma development. There is also evidence that there may be a link between asthma pathobiology and neuroinflammation, suggesting that GLP-1 and its analogs may regulate neuroinflammatory pathways that contribute to asthma pathogenesis. Interest is growing, though research remains limited, in how inflammation in the nervous system and lung might be linked. This review will explore how GLP-1R signaling could inhibit interdependent inflammation in both the lung and nervous system. This review will first focus on the inflammation that is known to exist in asthma, then pivot to the current state of neural regulation of asthma, and finally speculate on how GLP-1RA signaling could inhibit both neural and lung inflammation in asthma treatment.

## Introduction

Asthma is among the most prevalent chronic respiratory diseases and is characterized by airway inflammation [1] and recurrent exacerbations [2] that affect both children and adults. The prevalence is higher in developed high-income countries, though the burden is growing in low- and middle-income countries where healthcare is not as accessible and essential asthma medications are unattainable [3]. Asthma produces a considerable amount of impairment resulting from dyspnea and exercise limitations that negatively impact daily activities, thus lowering quality of life [3]. Asthma is often seen as an allergic disease primarily driven by type 2 inflammatory immune cells, including mast cells, basophils, CD4+ T-helper type 2 (Th2) cells, eosinophils, and group 2 innate lymphoid cells (ILC2s). When exposed to various environmental triggers, the body’s innate immune response produces alarmins, particularly interleukin-33 (IL-33), that are released primarily from airway epithelial cells and macrophages [4]. Other alarmin cytokines that augment Th2 responses include thymic stromal lymphopoietin (TSLP) and IL-25. These alarmins enhance the type 2 allergic inflammatory response by activating mast cells, Th2 cells, ILC2s, and basophils. This initiates these cells to produce cytokines such as IL-4, IL-5, IL-9, and IL-13, that can activate structural cells in the lungs, namely goblet cells and smooth muscle cells, initiating airway remodeling resulting in hyperresponsive airways [4]. Asthma is considered a heterogeneous condition with various phenotypes, typically categorized into allergic and non-allergic asthma [5]. Non-allergic asthma is also associated with airway neutrophilia, however, the airway inflammation that drives allergic asthma will be the primary focus of this review.

The most recurrent subtype of asthma is allergic asthma, impacting approximately 60% of all asthma patients and 50% of those with severe diseases [1]. Allergic asthma commonly begins in childhood as sensitization to airborne allergens typically occurs after age 2 [6, 7]. Its prevalence increases throughout childhood and adolescence, reaching its peak during the second decade of life. Various factors can trigger asthma exacerbations, such as viral infections, physical activity, exposure to irritants, allergen exposure in sensitized individuals, and consumption of nonsteroidal anti-inflammatory drugs (NSAIDs) [7]. Uncontrolled asthma is defined by symptom occurrence, the need for rapid relief medications, and exacerbation frequency that may necessitate treatment with corticosteroids [2]. Well-controlled asthma patients have fewer exacerbations and typically need only quick-relief medications, such as short-acting β2-agonists (SABAs). However, studies have indicated that asthmatic individuals often use quick-relief medications daily instead of long-term control medications. People with poor asthma control have an elevated risk of morbidity, highlighting the necessity for improved utilization of long-term control medications [2]. The use of biologic agents that target allergic inflammation has revolutionized the treatment of people who do not have full responses to the standard inhalational therapy mentioned above, yet there are still patients who have uncontrolled disease. This is particularly the case in obese persons with asthma, and novel treatment approaches in this population will be highlighted in this review.

Previous studies have observed a connection between metabolic dysfunction and asthma, where individuals with insulin resistance have increased asthma incidence [8, 9]. Obesity contributes to this issue, as it is linked with chronic, low-grade inflammation, which correlates with conditions such as insulin resistance, type 2 diabetes mellitus (T2DM), nonalcoholic fatty liver disease, and atherosclerosis [10]. Compared to lean and overweight individuals, patients with both asthma and obesity tend to experience a higher frequency of healthcare visits, exacerbation rates, and greater reliance on medications [8, 9]. This suggests insufficient symptom control, potentially indicating a link between the severity of asthma and metabolic disorders [8]. Notably, insulin resistance plays a crucial role as a common underlying mechanism linking asthma to various comorbidities, particularly T2DM [11]. Diabetes-related microangiopathy, caused by hyperglycemia, affects the lungs by damaging microvasculature [12]. This damage is due to the glycosylation of proteins like collagen and elastin in the connective tissue of the lungs that leads to cross-linking, making collagen more resistant to degradation, and potentially contributing to airway remodeling [11]. Epidemiological studies suggest that diabetic individuals experience heightened airway responsiveness and a reduction in forced expiratory volume in one second (FEV1) [11]. These associations underscore the interconnected roles of metabolic dysfunction and asthma severity, particularly as obesity has been established as a significant risk factor for asthma in recent years [13].

An American Thoracic Society workshop report identified obesity as a contributing risk factor for asthma across diverse demographic populations, indicating that asthma in obese individuals may reflect a distinct phenotype characterized by greater disease severity and reduced response to standard therapies. Further epidemiological studies emphasize that as body mass index (BMI) increases, so does the incidence of developing asthma, specifically in those with atopic diseases, such as allergic rhinitis, allergic asthma, or atopic dermatitis [10, 13]. These findings highlight the expanding population facing both asthma and obesity, signifying the need for treatment strategies that address the mechanical and inflammatory factors correlated with obesity [10]. The mechanical impacts of obesity on the lungs include reductions in functional residual capacity (FRC) and expiratory reserve volume (ERV), caused by increased abdominal pressure and chest wall fat compressing the lungs and narrowing airways. This results in increased airway resistance and responsiveness, potentially due to changes in airway smooth muscle (ASM) contractility. Additionally, obese individuals often experience greater breathlessness during bronchoconstriction due to elastic loads not being fully captured by spirometry, which could help account for the increased severity of symptoms observed in obese asthmatics than those in lean individuals. While these mechanical factors play a significant role, obesity-related chronic, low-grade inflammation also contributes to the pathophysiology of asthma. This inflammatory component, driven by immune system alterations and adipokine imbalances, is discussed in greater detail later in this paper [10]. There is an unmet need for new medical therapies, particularly among patients who have both obesity and asthma, who do not respond well to conventional inhaler treatments [8]. This issue is especially prevalent for severe asthma patients who fail to respond to high-dose inhaled glucocorticoid treatment, a condition known as “steroid-resistant (SR) asthma” [14]. There is experimental evidence that pharmacological treatments targeting insulin resistance, such as metformin and sulfonylureas [15, 16], may improve asthma management [8]. The glucagon-like peptide-1 receptor agonists (GLP-1RAs) are a group of medications that are U.S. Food and Drug Administration (FDA) approved for the stepwise treatment of T2DM. By enhancing insulin secretion and reducing glucagon secretion, this class of drugs reduced hyperglycemia along with decreasing the risk of cardiovascular and renal conditions, in addition to minimizing all-cause mortality rates in individuals with T2DM [8]. GLP-1RAs were more effective than metformin, resulting in quicker weight loss and improved glycemic control [8, 17]. While metformin indirectly promotes weight loss by activating GLP-1R, the pathways the GLP-1RA utilize have a more immediate effect in reducing gastric emptying and heightening levels of satiety [8]. GLP-1 is a part of the neuroendocrine peptide hormone family commonly known as incretins. GLP-1R is expressed in several organs such as the heart, kidneys, pancreas, gastrointestinal tract, lungs, and brain [4]. While it is mainly secreted into the bloodstream from intestinal cells, GLP-1 also influences metabolism through the gut-brain axis, specifically, small amounts of GLP-1 are synthesized in distinct neuronal populations in the hindbrain [18]. GLP-1 has an impact on various biological mechanisms, including improved insulin sensitivity, inhibition of glucagon synthesis, slowing gastric emptying [19], and reduced pancreatic β cell apoptosis, when it signals through its specialized G protein-coupled receptor (GPCR), GLP-1R [20]. GLP-1R activation provides anti-inflammatory, metabolic, cardiovascular, and neuroprotective properties [8].

Growing evidence indicates that the inflammation that occurs in asthma extends beyond the airways and is systemic [21], increasing pro-inflammatory cytokines that may negatively impact neurologic function. Patients with allergic asthma have elevated serum levels of tumor necrosis factor alpha (TNF-α), a pro-inflammatory cytokine, due to increased expression by mucosal mast cells [22, 23]. High levels of TNF-α leads to bronchial inflammation and airway damage [22]. Additionally, TNF-α contributes to blood-brain barrier (BBB) disruption and may be a mediator in inflammatory central nervous system (CNS) diseases [24]. Mast cells, known for their role in allergic inflammation, release a variety of mediators and cytokines in response to both immunoglobulin E (IgE)-dependent and nonimmunologic stimuli [23]. These cells are also located in the CNS, where they line the cerebral blood vessels and contribute to neuroinflammatory responses [25]. However, the influence and specific mechanisms of chronic or acute peripheral lung inflammation on neurofunctions remain unclear.

Chronic airway inflammation in asthma, characterized by elevated cytokine levels such as IL-4, IL-5, IL-13, and TNF-α [26], can contribute to systemic effects that, in turn, influence the CNS. This systemic inflammation, along with increased cytokine levels, not only impacts the CNS but also engages the peripheral nervous system (PNS), which is pivotal in driving both neuroinflammation and asthma pathology. The PNS mediates bidirectional communication between peripheral tissues and the CNS, influencing both immune responses and autonomic control [27]. The PNS is comprised of the autonomic nervous system (ANS) and the somatic nervous system (SNS). The ANS regulates the homeostasis of various internal organs and metabolic processes, including the respiratory system (lung and airways) and energy metabolism (e.g., glucose and lipid regulation). The ANS is further divided into the sympathetic and parasympathetic nervous systems [28], where the parasympathetic nervous system specifically regulates allergic reactions and chronic symptoms in asthma, primarily through effects on airway tone and mucus production [29]. It controls smooth muscle contraction and mucus secretion by releasing acetylcholine (ACh), which acts on muscarinic receptors (notably the M3 subtype) in the ASM and submucosal glands. In asthma, this pathway is often overactivated, leading to bronchoconstriction and increased mucus production, exacerbating asthma symptoms [29]. In allergic asthma, inflammation disrupts normal parasympathetic feedback mechanisms. Eosinophils, common immune cells in asthma, release proteins like eosinophil major basic protein (MBP), which antagonize the M2 muscarinic receptors on parasympathetic nerves. This disruption prevents the usual negative feedback mechanisms that limit ACh release, resulting in excessive bronchoconstriction and airway hyperreactivity [29]. Muscarinic receptors are a type of GPCRs with 5 subtypes (M1–M5) and are activated by the neurotransmitter ACh [30]. Both muscarinic receptors and GLP-1Rs are GPCRs, but they function in different physiological contexts: muscarinic receptors are primarily involved in the ANS, while GLP-1Rs play a key role in metabolic control. Furthermore, GLP-1R activation is associated with the modulation of inflammation and immune responses [11], processes that are also regulated by muscarinic receptor activity in inflammatory conditions, such as asthma [29]. This connection hints at potential interactions between the muscarinic receptors and the GLP-1 signaling pathways, particularly in scenarios involving airway inflammation and metabolic diseases. Recent studies have reported connections between lung diseases, such as chronic obstructive pulmonary disease (COPD) and asthma, with depression, anxiety, and cognitive decline/dementia [1]. For example, chronic intranasal administration of house dust mite (HDM) antigen led to impaired spatial memory and depression-like behaviors in mice. HDM antigens commonly produce allergic lung inflammation, and as such, are commonly used in mice to model human asthma [1]. Immune-derived cytokines, specifically IL-1β and TNF-α, mediate communication between the immune system and brain [31], and these cytokines were measured in the serum of the HDM-sensitized mice. HDM-sensitized mice showed significantly higher levels of vascular endothelial growth factor (VEGF) and pro-inflammatory cytokines [IL-β, IL-2, IL-4, IL-5, IL-13, interferon (IFN)-γ] compared to control mice, indicating an increased inflammatory response. IL-1β is typically known to affect paracellular barriers that lead to BBB disruption, while VEGF enhances BBB permeability [1]. Elevated serum VEGF concentrations were found in asthmatic patients and may be a biomarker for exacerbations [32]. A positive correlation was also established between serum VEGF and depression severity asthma in recent studies [33]. These findings suggest that allergen-induced inflammation may result in systemic production of these pro-inflammatory cytokines, potentially increasing the risk for depression [1].

In addition to its association with depression, asthma is strongly linked to anxiety, a common neurological condition characterized by excessive worry and fear. Recent studies have revealed that individuals with asthma are nearly three times as likely to develop anxiety disorders compared to healthy individuals [34]. Severe asthma affects a small percentage of the asthma population, around 3–8%, but it severely negatively impacts their quality of life. This is due to the high frequency of acute exacerbations and the presence of multiple comorbid conditions, which together contribute to a more significant burden on affected individuals [35]. Neuroinflammation in asthma, driven by elevated type 2 inflammatory cytokines, plays a vital role in anxiety-like behaviors. The amygdala and medial prefrontal cortex (mPFC) are key brain regions responsible for anxiety processing networks. In a recent study, an asthma-induced rat model showed that chronic airway inflammation enhanced microglial and astrocytic activation in these specific brain regions. This activation, potentially mediated by peripheral nerve signaling or inflammatory factors crossing the BBB, disrupts neural activity and connectivity in anxiety-related circuits, such as the mPFC-amygdala axis. Further evidence suggests that inhaled corticosteroids may alleviate these neuroinflammatory changes, improving functional connectivity in anxiety circuits and offering therapeutic potential for anxiety in asthma patients [34].

GLP-1RAs have exhibited anti-inflammatory, neurotrophic, and neuroprotective properties in preclinical models of neurodegenerative disorders. Consequently, there is significant promise for repurposing these drugs as treatments for neurodegenerative diseases in people [36]. By examining the connection between asthma and neuroinflammation, we can identify potential therapeutic strategies for mitigating the damaging effects of asthma on the brain, where GLP-1RAs present novel opportunities to target neuroinflammation in conjunction with asthma.

## Neural pathogenesis of asthma

The neuropathological impact of asthma on the brain is not completely understood, and its magnitude remains unclear despite various evidence from epidemiological, animal model, and longitudinal studies [1, 37–41]. Various risk factors have been linked to neurodegenerative diseases, neuroinflammation being the primary driver, along with genetic, environmental, and lifestyle factors. Emerging research suggests a potential connection between asthma pathobiology and neuroinflammation, raising the possibility that GLP-1 and its analogs may play a role in modulating neuroinflammatory pathways implicated in asthma development and progression [42]. Asthma has numerous etiologies and molecular phenotypes (neutrophilic, eosinophilic, and paucigranulocytic that characterize airway inflammation) [38]. Airway inflammation may not only affect the airways but could also have systemic manifestations. The exploration of whether airway inflammation could damage brain health, beyond known associations with depression, is still a topic yet fully investigated. Several population studies suggest there is a greater risk for persons with asthma to develop dementia, specifically if the individuals experience frequent or severe exacerbations [37, 40, 41, 43]. Animal studies also showed the connection between airway inflammation and neuroinflammation [37, 44]. For instance, allergen exposure in an asthma mouse model led to microglial cell activation, stimulation of pro-inflammatory cytokine expression, and damaging structural changes in the same brain regions associated with dementia and Alzheimer’s disease (AD) [38, 44]. Changes in brain function in relation to airway inflammation have been established, but studies examining the broader health impacts on the brain have yet to be supported [37].

The prevalence of neurodegenerative diseases has increased and is often associated with chronic neuroinflammation. This connection is distinctly intertwined with obesity, a canonical precursor to various metabolic disorders, including T2DM, hypertension, and hyperlipidemia. Obesity, often resulting from overnutrition, leads to the expansion of white adipose tissue (WAT) [45]. As the main fat-storage site and largest endocrine organ, WAT systemically secretes adipokines and cytokines [46]. Early studies have indicated that systemic inflammation associated with obesity and high-fat diets (HFDs) in obese humans and HFD-fed rats results in higher levels of circulating pro-inflammatory cytokines (IL-1, IL-6, and TNF-α) and increased macrophage infiltration into the WAT when compared to the controls [47]. The connection between WAT and asthma has been minimally explored, but there may be a potential link based on the activation of similar pro-inflammatory cytokines. Furthermore, recent research reveals that obesity can also stimulate central inflammation. For instance, in murine models, HFD was shown to induce hypothalamic inflammation, characterized by increased pro-inflammatory cytokine expression, activation of transcription factor NF-κB, microglial activation, and increased levels of local cytokines [47, 48]. When there becomes an overproduction of these proinflammatory cytokines, the risk of developing chronic neuroinflammation, leading to neurodegenerative diseases, such as AD, increases. This intricate exchange emphasizes the extensive impact of metabolic health on brain function and health [45].

Neuroinflammation and neurodegeneration can begin to develop when microglia and astrocytes become activated. Microglia perform a critical role in governing neuroinflammation within the CNS. They present a dual functionality, transitioning between the inflammatory “M1” state and the anti-inflammatory “M2” state. Pro-inflammatory factors like lipopolysaccharide (LPS) and IFN-γ, are elevated in obesity and trigger microglia to differentiate into the neurotoxic M1 type. Conversely, microglia can then switch over to a beneficial and neuroprotective M2 state in response to IL-4 and IL-10 signaling [45]. Furthermore, astrocytes, the most abundant cells in the brain, serve as key regulators of brain glucose metabolism and neuronal functions [49]. Astrocytes are converted from a resting form to a reactive form by activated microglia secreting IL-1α, TNF-α, and C1q, contributing to neurodegenerative diseases like AD and Parkinson’s disease (PD) [50]. Recent studies have also revealed the versatile role of astrocytes, not only in balancing glucose and energy responses to hormones like leptin and insulin but also in modulating glucose transport, thereby affecting behavioral responses [41]. Hence, chronic microglial stimulation, leading to reactive astrocytes, can likely promote neurotoxicity and neurodegeneration [50].

**Figure 1.**
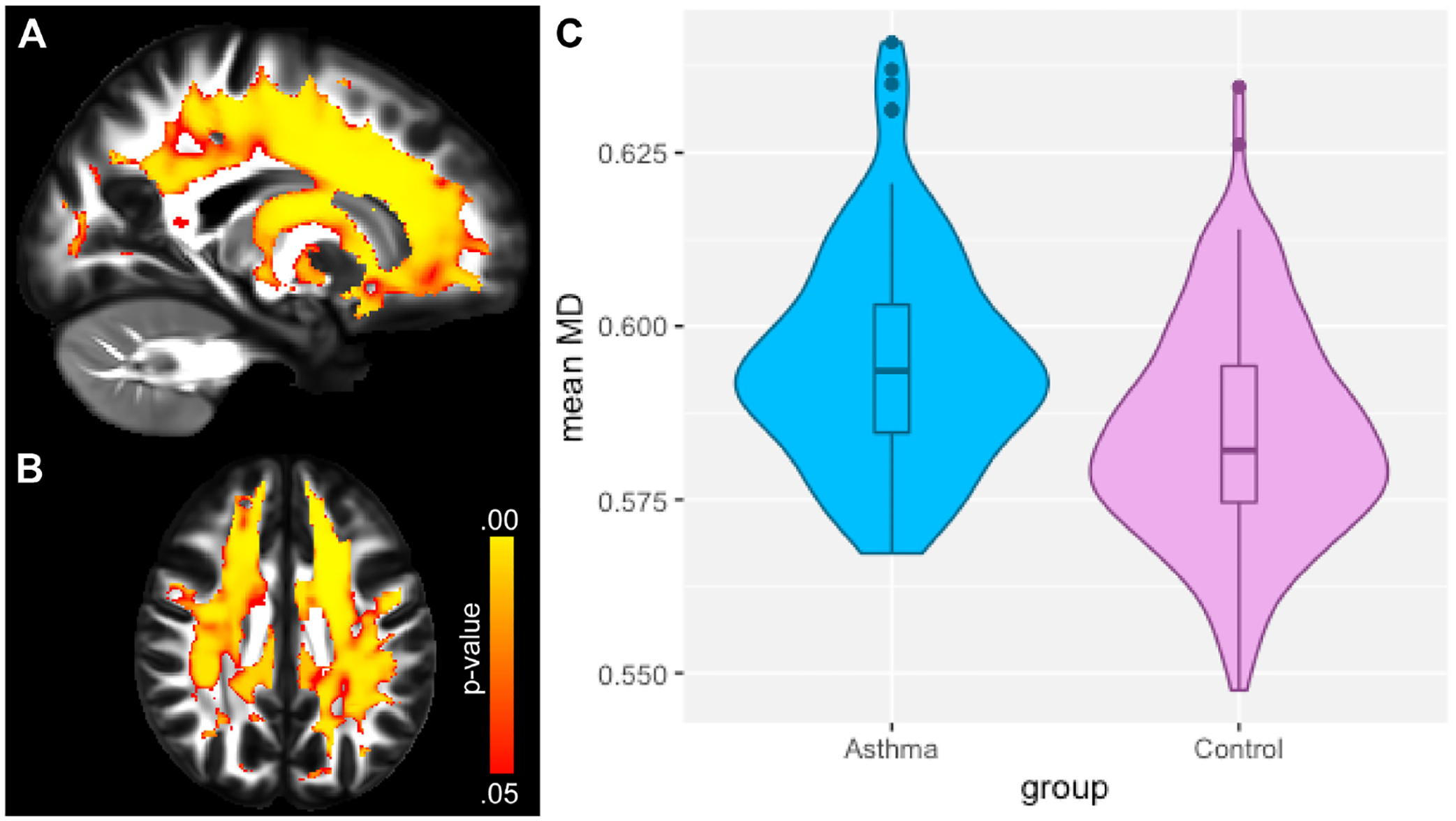
Neuroimaging of white matter microstructure demonstrates differences between individuals with asthma and healthy controls. (**A**) and (**B**) display sagittal and axial views of the brain, highlighting regions with significant group differences using a yellow-orange color scale. (**C**) presents the distribution of mean diffusivity (MD) values for each group, averaged over brain regions where MD was significantly higher in the asthma group. This visualization emphasizes the structural brain differences associated with asthma compared to controls *Note*. Reprinted with permission from “Neuroimaging and biomarker evidence of neurodegeneration in asthma” by Rosenkranz MA, Dean DC 3rd, Bendlin BB, Jarjour NN, Esnault S, Zetterberg H, et al. J Allergy Clin Immunol. 2022;149:589–98.e6 (https://doi.org/10.1016/j.jaci.2021.09.010). © 2021 American Academy of Allergy, Asthma & Immunology.

**Figure 2.**
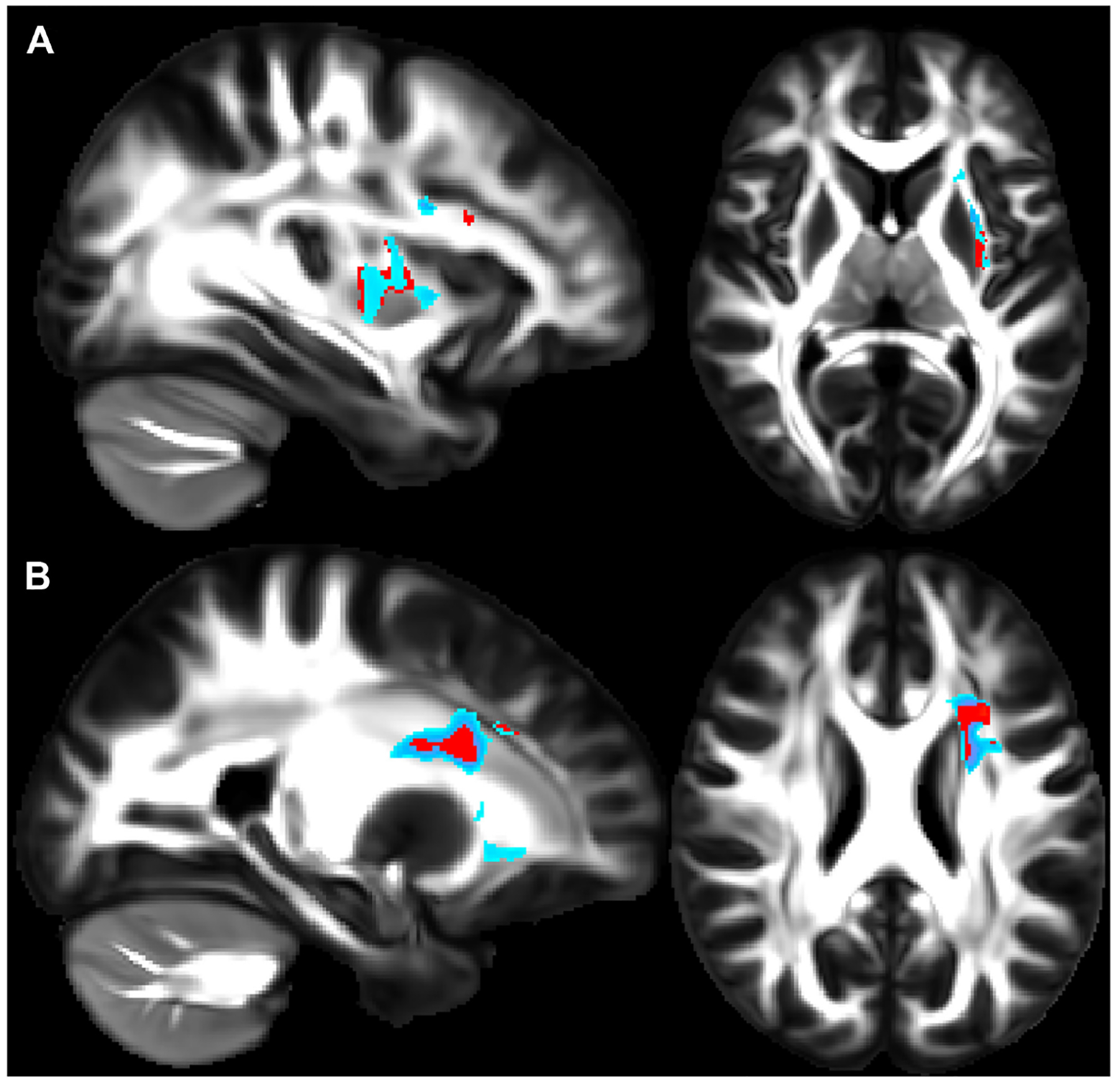
Higher asthma severity reveals weaker white matter integrity. (**A**) and (**B**) show sagittal (left) and axial (right) views that illustrate where asthma severity is significantly correlated with white matter microstructure changes. Regions in red highlight areas associated with overall changes in white matter microstructure across all diffusion-weighted imaging (DWI) metrics, while regions in blue indicate areas with increased mean diffusivity (MD) values. In panel A, affected regions include the inferior fronto-occipital fasciculus, superior longitudinal fasciculus, and uncinate fasciculus. In panel B, the highlighted regions include fibers in the superior longitudinal fasciculus, anterior thalamic radiation, and uncinate fasciculus *Note*. Reprinted with permission from “Neuroimaging and biomarker evidence of neurodegeneration in asthma” by Rosenkranz MA, Dean DC 3rd, Bendlin BB, Jarjour NN, Esnault S, Zetterberg H, et al. J Allergy Clin Immunol. 2022;149:589–98.e6 (https://doi.org/10.1016/j.jaci.2021.09.010). © 2021 American Academy of Allergy, Asthma & Immunology.

**Figure 3.**
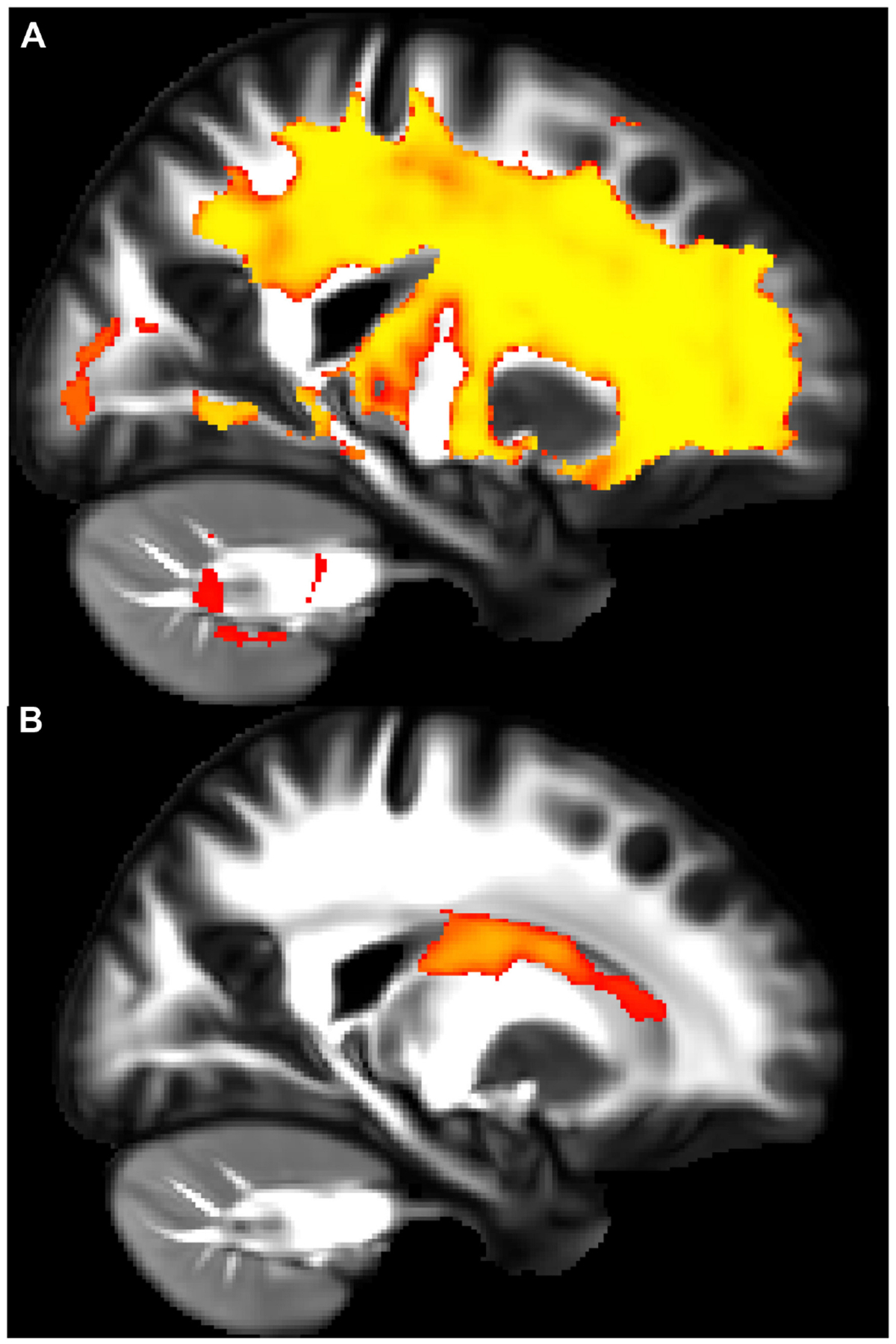
Elevated plasma biomarker levels are associated with white matter quality. (**A**) and (**B**) show a sagittal view showing regions where plasma biomarker concentrations negatively correlate with neurite density index (NDI) values. In panel A, the yellow areas show the glial fibrillary acidic protein (GFAP) concentration that demonstrates a significant negative association with NDI, indicating neuroinflammation. In panel B, the red areas show the neurofilament light chain (NfL) concentration that reveals a similar negative relationship with NDI, indicating neurodegeneration. The affected regions primarily include fibers within the corona radiata and internal capsule, such as the corticospinal tract and thalamic radiations *Note*. Reprinted with permission from “Neuroimaging and biomarker evidence of neurodegeneration in asthma” by Rosenkranz MA, Dean DC 3rd, Bendlin BB, Jarjour NN, Esnault S, Zetterberg H, et al. J Allergy Clin Immunol. 2022;149:589–98.e6 (https://doi.org/10.1016/j.jaci.2021.09.010). © 2021 American Academy of Allergy, Asthma & Immunology.

A recent study showed how asthma was connected to notable deleterious modifications in white matter in the brain, resembling the extent of that which is seen in neurodegenerative diseases. Across patients aged 18–73 years old, patients with asthma (*n* = 111) showed defects in white matter confirmed by high levels of plasma biomarkers such as neurofilament light chain (NfL), indicating neurodegeneration, and glial fibrillary acidic protein (GFAP), indicating neuroinflammation, compared to healthy controls (*n* = 135) [37]. Diffusion-weighted MRI (dMRI) and mean diffusivity (MD) were used to compare white matter microstructure between the asthmatic and control groups. dMRI assesses localized brain microstructural change, while MD measures the diffusion of water molecules in the white matter areas of the brain. MRI scans were acquired under normal conditions, not during asthma exacerbations, ensuring consistency and reduced variability from acute inflammatory or physiological changes [37]. Figure 1A and B highlight overall group differences with yellow-orange areas indicating significant differences in white matter microstructure between the groups. Figure 1C reveals there were higher MD values in the asthma group versus the control group, suggesting alterations or deterioration in the white matter microstructure. Findings in Figure 2A and B highlight red regions where asthma severity correlates with reduced white matter integrity (shown as blue regions of higher MD). These regions indicate that individuals with severe asthma have compromised white matter, including deteriorated myelinated axons. This suggests potential damage or dysfunction in the protective myelin sheaths that surround the nerve fibers in the white matter. Notably, elevated plasma biomarker concentrations are linked to reduced white matter quality (Figure 3A and B), showing brain regions where GFAP and, to a smaller extent, NfL concentrations are amplified, affecting neurite density reduction and neuronal health processes. Overall, these differences in dMRI metrics were more pronounced among participants with severe asthma [37]. The brain regions associated with these significant findings include the inferior fronto-occipital fasciculus that is a key white matter tract in the ventral brain that connects the frontal lobe to the parietal, temporal, and occipital areas, involved in language processing, semantic understanding, response inhibition, and action regulation [51]; the superior longitudinal fasciculus is an extensive fiber bundle system, acting as a communication bridge linking regions involved in language, spatial awareness, and cognitive control [52]; corticospinal tract which is a major neural pathway responsible for facilitating voluntary movement [53]; the corona radiata serves as an essential white matter tract that connects the thalamus and anterior cingulate cortex to the lateral prefrontal cortex [54]; and the internal capsule fibers are a bidirectional pathway that facilitates communication between the cerebral cortex and subcortical structures, transmitting motor and sensory information [37, 55].

Elevated GFAP expression levels are a characteristic of reactive astrocytes that are used together with NfL as markers of advancement of neurodegenerative diseases. Reactive astrocytes play a vital role in preserving brain health; however, their presence can signify neuroinflammation leading to neuronal degeneration, enhancement of microvascular permeability, and increased inflammation through interactions with microglia [37]. Another significant finding in relation to decreased white matter integrity is slower processing speeds, a standard form of measurement of cognitive function. dMRI’s revealed slower processing speeds observed in asthmatics in areas with deteriorated white matter quality, suggesting accelerated cognitive decline. These results revealed an asthma association with glial activation, neuroinflammation, and neurodegenerative processes, which may decrease cognitive function independently of normal aging [37].

**Figure 4.**
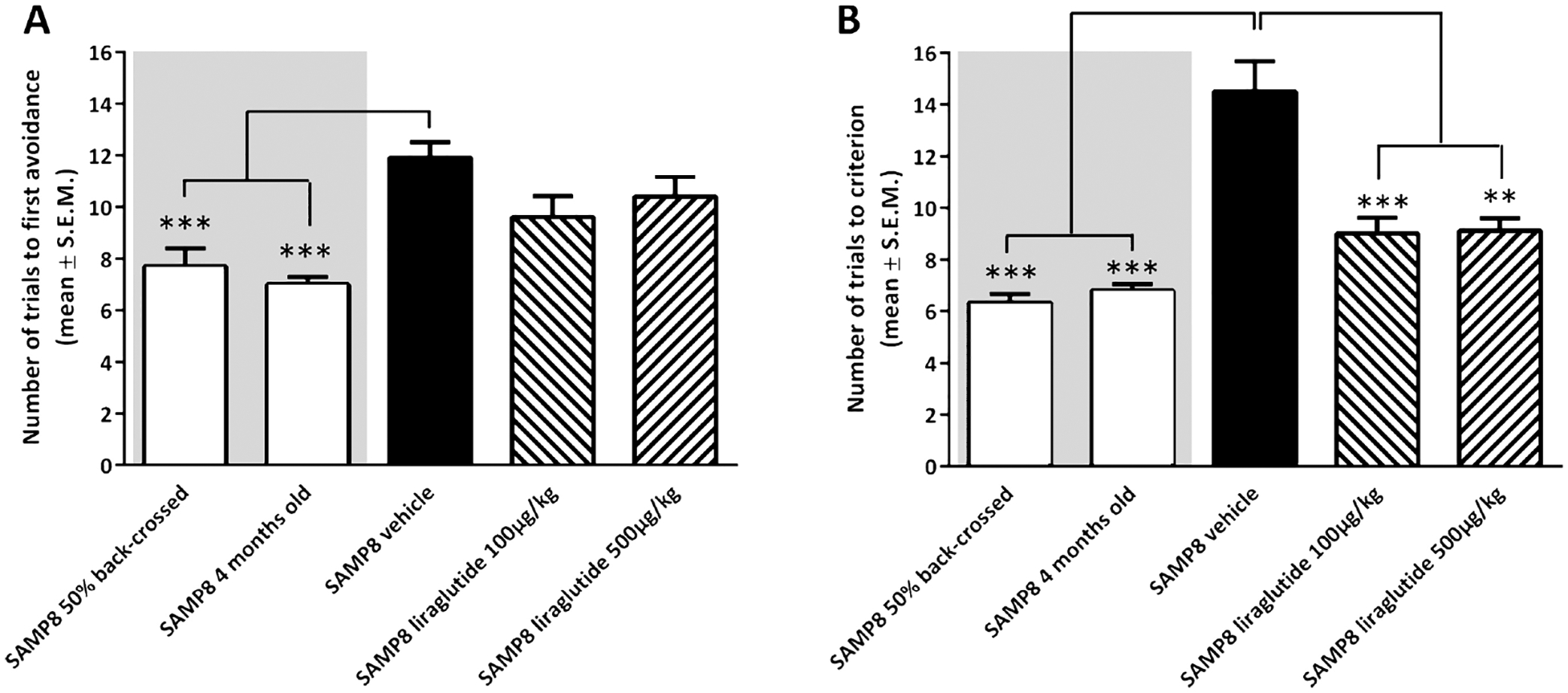
A T-maze test was performed to assess memory acquisition and retention function in senescence-accelerated mouse prone 8 (SAMP8) mice. (**A**) shows memory acquisition, measured as the number of trials required to make one active avoidance. (**B**) portrays memory retention, evaluated one week later by the number of trials needed to achieve five action avoidances in six consecutive trials. Vehicle-treated 10-month-old SAMP8 mice exhibited impaired memory compared to controls, including four-month-old untreated SAMP8 mice and 50% back crossed vehicle-treated SAMP8 mice. Notably, long-term liraglutide treatment restored memory retention in an older sample of eight mice two levels comparable to younger controls. **: *P* < 0.01; ***: *P* < 0.001 *Note*. Reprinted from “The GLP-1 Receptor Agonist Liraglutide Improves Memory Function and Increases Hippocampal CA1 Neuronal Numbers in a Senescence-Accelerated Mouse Model of Alzheimer’s Disease” by Hansen HH, Fabricius K, Barkholt P, Niehoff ML, Morley JE, Jelsing J, et al. J Alzheimers Dis. 2015;46:877–88 (https://doi.org/10.3233/JAD-143090). CC BY-NC.

The mechanisms through which GLP-1 and its agonists influence brain function are still being defined. Nonetheless, numerous studies have highlighted specific roles that GLP-1RAs play within the brain, along with their involvement in regulating neuronal structure and function, while providing neuroprotective properties. Specifically, GLP-1RAs activate the GLP-1R signaling pathway by crossing the BBB and in various extrahypothalamic regions, such as the cortex, thalamus, hypothalamus, brainstem, and hippocampus [45, 56]. GLP-1, naturally produced in the hindbrain, has an influence on diverse brain regions, with a notable impact on the hypothalamus, where it regulates eating patterns and body weight by decreasing appetite and food consumption, resulting in weight loss. Remarkably, GLP-1RAs can effectively modulate these brain functions even when the GLP-1R is absent in this specific hypothalamic region, suggesting the drug may have receptor-independent actions [49]. A study using liraglutide, a long-acting GLP-1RA, sought to assess its influence on memory function and hippocampal neuron numbers in senescence-accelerated mouse prone 8 (SAMP8) mice, a model for age-related sporadic AD without amyloid plaques [57]. This model allowed examination of the drug’s pro-cognitive effects in early AD stages before irreparable late-stage plaque-associated toxicity develops. The study included young 4-month-old SAMP8 mice, and age-matched, genetically modified SAMP8 mice with normal memory, to control for age-dependent memory decline. The data in Figure 4 shows the number of trials needed to reach a single active avoidance, with the liraglutide-treated SAMP8 mice exhibiting enhanced and restored memory retention (Figure 4A). A T-maze test with foot-shock avoidance was used to measure memory retention and examine significant differences in memory performance between AD mice and the control mice (Figure 4B). A week later, memory retention was evaluated by continuing training until the mice achieved five active avoidances in six continuous trial runs. These results revealed that the liraglutide-treated SAMP8 mice required considerably fewer trials compared to the vehicle-dosed SAMP8 control mice. This indicates that the AD mice treated with the GLP-1RA displayed memory function comparable to the genetically modified mice with regular memory function, both showing significant improvements over the vehicle-dosed AD SAMP8 mice (Figure 4B) [57].

The results from this study exhibit GLP-1RA’s potential in reducing neuroinflammation and associated disorders by reducing pro-inflammatory cytokines, improving neuronal survival, and preserving BBB integrity [57]. Another study explored whether pegylated exendin-4, long-acting GLP-1RA (NLY01), a CNS-penetrating, long-acting GLP-1RA, could reduce AD symptoms by inhibiting microglial activation and inflammatory mediators, thereby preventing astrocyte reactivity. In AD mouse models with intact GLP-1R, NLY01 selectively blocked microglial stimulation and the production of reactive astrocyte inducers (TNF-α, C1q, and IL-1α), successfully preserving neurons [50]. NLY01 also significantly diminished pro-inflammatory cytokine (TNF-α, IL-1α, IL-1β, and C1q) expression in the hippocampus, preventing the stimulation of reactive astrocytes. When examining AD mouse models in which GLP-1R was knocked out, NLY01 was unsuccessful in blocking microglial activation or production of reactive astrocyte inducers. This establishes that NLY01’s inhibitory effects are primarily facilitated through the GLP-1R. These findings suggest that GLP-1RAs, like NLY01, can lessen AD pathology by targeting neuroinflammation related to microglial activation and reactive astrocytes, thus restoring memory functions and offering neuroprotective benefits [50]. As our understanding of neuroinflammation’s role in conditions like AD and cognitive disorders advances, GLP-1RAs reveal prospective therapeutic options for these complex challenges. It will be interesting to determine if NLY01 reduces allergic lung inflammation similar to its effects on inhibiting neuroinflammation described above.

All in all, inflammation concomitant with asthma activates inflammation in the CNS, exposing related brain regions to potential damage, eventually advancing cognitive impairment. Established from this data, brain health is impacted by deteriorated white matter integrity in the presence of asthma. To further determine if asthma is a factor leading to the development of neuroinflammation and neurodegeneration, future long-term studies are required to identify if asthma definitively plays a role in accelerating the risk of white matter damage that could eventually turn into neurological deficits [37].

## How GLP-1RA might reduce inflammation to reduce allergic inflammation

**Figure 5.**
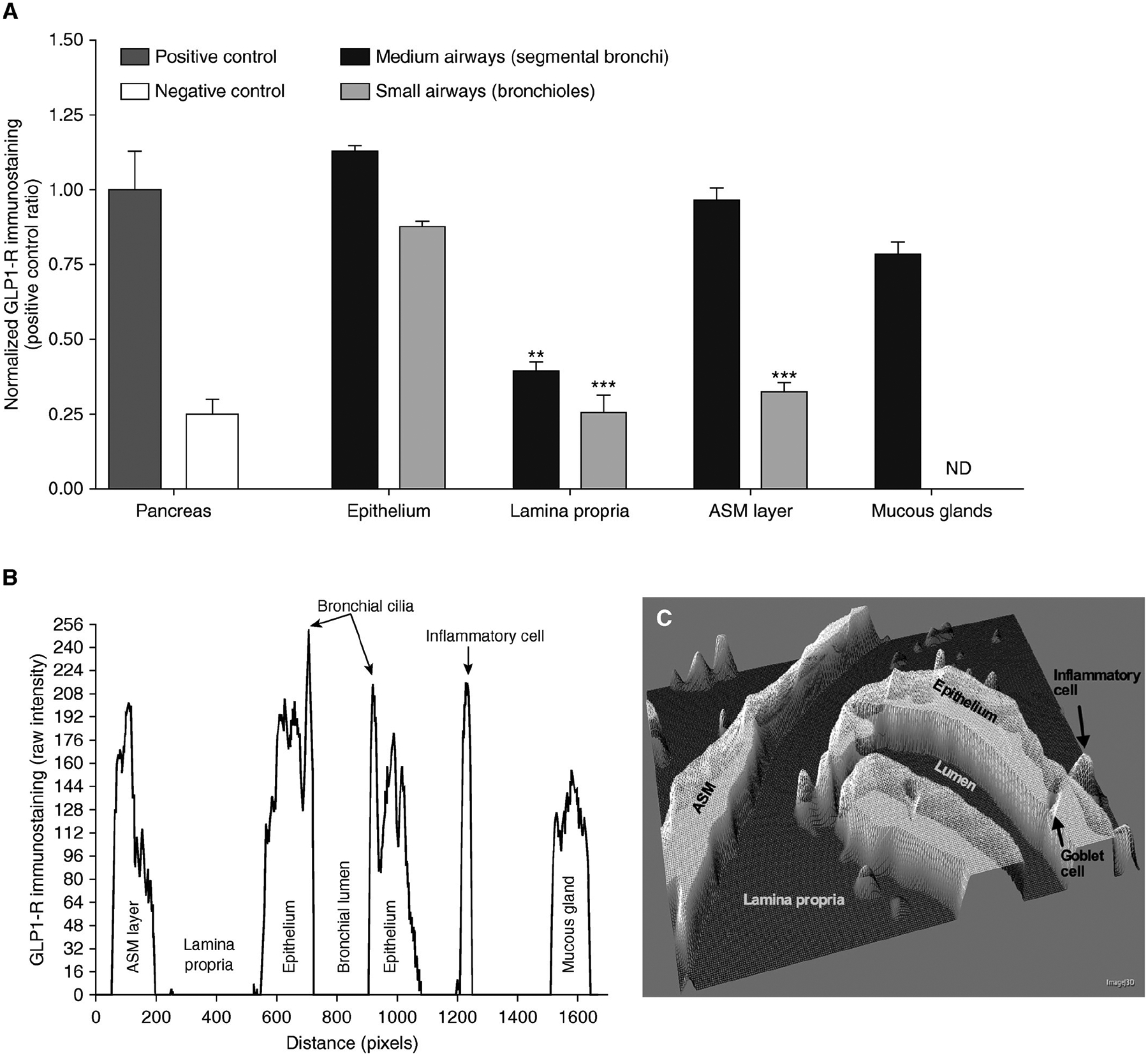
The Glucagon-like peptide-1 receptor (GLP-1R) is expressed in human bronchi. (**A**) displays a bar graph quantifying normalized GLP-1R immunoreactivity in medium and small bronchi, with pancreatic tissue serving as a positive control and untreated tissue as a negative control. (**B**) presents raw immunostaining data for GLP-1R in a bronchial section. (**C**) shows an interactive three-dimensional surface plot, highlighting GLP-1R expression. Results are based on samples from five different subjects. ASM: airway smooth muscle; ND: not detectable; **: *P* < 0.01; ***: *P* < 0.001 *Note*. Reprinted with permission from “Glucagon-Like Peptide 1 Receptor: A Novel Pharmacological Target for Treating Human Bronchial Hyperresponsiveness” by Rogliani P, Calzetta L, Capuani B, Facciolo F, Cazzola M, Lauro D, et al. Am J Respir Cell Mol Biol. 2016;55:804–14 (https://doi.org/10.1165/rcmb.2015-0311OC). © 2016 by the American Thoracic Society.

GLP-1R protein expression has been observed to be considerably enhanced in the lung, specifically in the lung epithelial and endothelial cells, suggesting it could play a vital role in lung function [4]. In asthma, airway obstruction is generally reversible; however, chronic inflammation can potentially result in irreversible airway remodeling, the components of which include smooth muscle hypertrophy, mucus metaplasia, and collagen deposition [58]. Bronchial hyperresponsiveness (BHR) is a hallmark of chronic asthma, is linked with disease severity, and is a consequence of changes in ASM tone [11, 59]. Previous studies have highlighted the potential role of GLP-1R activation in modulating airway function. In ex vivo models, exendin-4 (a GLP-1RA), induced a bronchorelaxant effect when the GLP-1R was activated. Notably, this effect is epithelium-independent and involves the activation of the cAMP-PKA signaling pathway, which mediates ASM relaxation and counteracts BHR [11]. A recent study treating ex vivo isolated human bronchi with exendin-4 demonstrated a positive GLP-1R immunostaining pattern in key airway structures, including the epithelium, bronchial cilia, mucous glands, inflammatory cells, and the ASM of medium-sized bronchi (Figure 5A). However, both the lamina propria and ASM of small bronchi exhibited lower immunoreactivity compared to the positive control, which consisted of pancreatic tissues (Figure 5B). Furthermore, GLP-1R expression was visualized through three-dimensional surface plots (Figure 5C), revealing its widespread distribution within the bronchial tissue, with differences in intensity seen in different parts of the lung [11]. Importantly, exendin-4 reduced GLP-1R overexpression in sensitized bronchi, suggesting a feedback mechanism that regulates receptor activation and prevents overstimulation. In addition to modulating ASM tone, GLP-1R activation may also reduce pro-inflammatory cytokine release, contributing to an anti-inflammatory effect that could be beneficial for asthma management. These findings support the notion that GLP-1R could play a direct role in lung function, potentially offering a new avenue for asthma treatment [11].

Obesity and asthma have been linked through chronic systemic inflammation driven by adipokines such as leptin, adiponectin, TNF-α, monocyte chemotactic protein-1 (MCP-1), and IL-6. Adipokines, produced by adipocytes in WAT, regulate both the innate and adaptive immune system by attracting and activating leukocytes and monocytes, leading to neutrophilic airway inflammation in obese asthmatics [60]. Leptin, which rises in direct correlation with obesity, promotes proinflammatory responses by activating immune cells and enhancing cytokine production, while adiponectin, which has decreasing levels with obesity, has anti-inflammatory properties and may reduce airway hyperreactivity. The imbalance between these adipokines contributes to systemic inflammation and may influence asthma severity [10].

WAT, the primary energy storage for the body, plays a central role in systemic inflammation. In obesity, inflamed and dysfunctional adipocytes within WAT release pro-inflammatory cytokines (TNF-α, IL-6, IL-8, and MCP-1) and attract immune cells such as macrophages, mast cells, and lymphocytes, further amplifying the immune response [60, 61]. Notably, WAT from obese individuals, both in human and mice studies, contained a higher number of mast cells, macrophages, and lymphocytes compared to the lean groups. These inflammatory cells in WAT produce cytokines, growth factors, chemokines, and proteases, contributing to systemic inflammation and altering macrophage activity [10, 62]. Weight gain initiates a phenotypic change in WAT, resulting in inflamed and dysfunctional adipocytes. These inflammatory cells exacerbate systemic and airway-specific inflammation, disrupting adipose tissue function and impacting other organs [46]. Obesity-associated leptin dysregulation also alters alveolar macrophage activity and leukotriene production, which may explain why obese asthmatics respond differently to corticosteroids but benefit from leukotriene modifiers. These inflammatory changes highlight how obesity exacerbates asthma severity and impacts treatment responses [10], suggesting that both conditions may represent manifestations of general systemic inflammation of the immune system [61].

Given that GLP-1RAs are used for obese individuals to help reduce weight and associated comorbidities, their potential benefits for asthma are worth considering. Evidence increasingly shows that obesity exacerbates asthma through the disruption of lipid metabolism and alterations in the endocrine functions of adipose tissue [63]. For instance, the adipokine leptin, which regulates the immune response, plays a role in the pathology of asthma and is distributed through the lungs. Obesity is linked to elevated leptin levels and leptin resistance, which impairs the hormone’s ability to regulate satiety. This impacts the hypothalamus, impairing its ability to control appetite and leading to increased food intake [63]. Studies using mild obese asthma mouse models have demonstrated that obesity prompts changes in adipokine secretion, such as leptin, adiponectin, and VEGF, yet there was no direct effect on airway hyperreactivity and lung inflammation development [14]. However, in mouse models of allergic airway disease, the obese mice developed more severe asthma conditions compared to the non-obese mice, suggesting that obesity worsens asthma through changes in adipokine secretion. Examining this connection further, another study using an obese asthma mouse model found that elevated leptin levels worsened airway inflammation by promoting inflammation through extracellular signal-regulated kinase (ERK) 1/2, p38 mitogen-activated protein kinase (p38-MAPK), MAPK kinase (MEK) 1/2, and NF-κB signaling pathways, pathways that are targeted to enhance inflammatory responses [14]. The links between chronic systemic inflammation driven by adipokines and the exacerbation of asthma symptoms in obese individuals highlight the need for a deeper understanding of the underlying mechanisms at play.

Overall, these findings promote the awareness that asthma may enhance the risk of developing brainrelated conditions, along with highlighting the positive impact GLP-1RAs have on these conditions independently. With the aim of advancing our understanding of the therapeutic potential of GLP-1RAs in addressing neuroinflammation and asthma, a clearer path to improved treatment methods for affected individuals is necessary. Various preclinical studies have established that allergic and viral-induced airway inflammation can be inhibited through the GLP-1R signaling pathway in both lean and obese murine models. Clinical studies have demonstrated in adult asthmatics that GLP-1RAs, liraglutide and semaglutide, reduce levels of serum periostin, a biomarker for airway inflammation and remodeling, that when compared with other diabetic medications, decreased the risk of asthma exacerbations [8, 64]. Furthermore, a new clinical trial is underway to determine if the GLP-1R signaling pathway influences airway inflammation in obese asthmatics, known as the GLP-1R Agonist in the Treatment of Adult, Obesity-related, Symptomatic Asthma (GATA-3) study. It is the first randomized, double-blind clinical trial investigating GLP-1RA’s impact on asthma. Results from this study could help expand the understanding of the therapeutic potential of a GLP-1RA for the treatment of asthma, within the context of obesity, by evaluating the direct impact it has on the airway and offering a therapeutic alternative for patients that no longer respond to traditional asthma treatment.

## Conclusions

**Figure 6.**
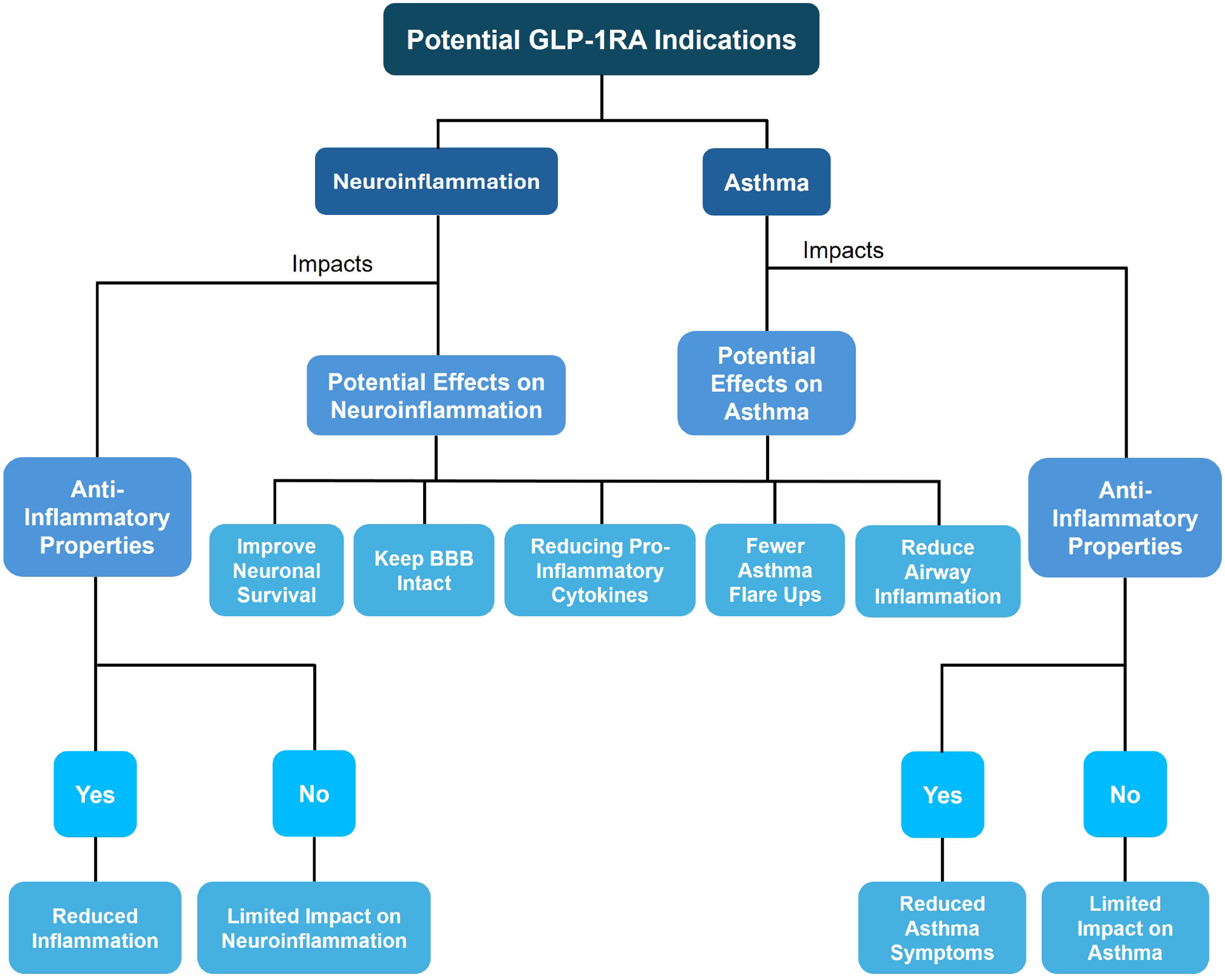
A schematic of the overall potential glucagon-like peptide-1 receptor agonist (GLP-1RA) indications and its connection to neuroinflammation and asthma. BBB: blood-brain barrier

The complex relationship between asthma, neuroinflammation, and the possible role of GLP-1RAs in reducing neuroinflammatory processes creates a promising path for additional research and therapeutic developments. While it is evident that asthma can induce systemic effects throughout the body, the precise mechanisms are not well known. By utilizing and understanding these various findings, future research might focus on examining the possibility that GLP-1RA drugs reduce pro-inflammatory cytokine responses in asthma and in the CNS, as this class of medications offers a potential positive effect in both diseases independently (Figure 6). Not only could this class of drugs provide beneficial protective properties for the brain, such as maintaining the stability of the BBB and improving neuronal survival, which is vital given the possible neuroinflammatory effects of chronic asthma, but it also may reduce airway inflammation. Additionally, the ability of GLP-1RA to regulate the complex interactions between the peripheral and central inflammatory pathways could also be valuable in determining whether there are indeed neuroprotective properties of this class of medications. GLP-1RAs have clearly defined benefits in metabolic and cardiovascular disorders, and the possibility that they could be beneficial in asthma and neurologic diseases could decrease the use of additional medications and improve quality of life.

Further longitudinal clinical investigations are needed to assess GLP-1RAs as a therapeutic option for reducing the risk of neuroinflammation in asthma. As this field of study progresses, such studies may provide insights into novel treatment methods for asthma patients, not only focusing on their respiratory symptoms but also their overall well-being, including brain health.

## Abbreviations

ACh: acetylcholine
AD: Alzheimer’s disease
ANS: autonomic nervous system
ASM: airway smooth muscle
BBB: blood-brain barrier
CNS: central nervous system
dMRI: diffusion-weighted MRI
GFAP: glial fibrillary acidic protein
GLP-1: glucagon-like peptide-1
GLP-1R: glucagon-like peptide-1 receptor
GLP-1RAs: glucagon-like peptide-1 receptor agonists
GPCR: G protein-coupled receptor
HDM: house dust mite
HFDs: high-fat diets
IL-33: interleukin-33
MD: mean diffusivity
NfL: neurofilament light chain
PNS: peripheral nervous system
SAMP8: senescence-accelerated mouse prone 8
T2DM: type 2 diabetes mellitus
Th2: T-helper type 2
TNF-α: tumor necrosis factor alpha
VEGF: vascular endothelial growth factor
WAT: white adipose tissue
